# Sex-specific association between carotid atherosclerosis and fundus arteriosclerosis in a Chinese population: a retrospective cross-sectional study

**DOI:** 10.1186/s40001-023-01508-6

**Published:** 2023-11-15

**Authors:** Chunxing Liu, Xiaolong Yang, Mengmeng Ji, Xiaowei Zhang, Xiyun Bian, Tingli Chen, Yihan Li, Xing Qi, Jianfeng Wu, Jing Wang, Zaixiang Tang

**Affiliations:** 1 Department of Laboratory, Hua Dong Sanatorium, Wuxi, 214065 China; 2 Department of Ophthalmology, Hua Dong Sanatorium, Wuxi, 214065 China; 3 Department of Nursing, Hua Dong Sanatorium, Wuxi, 214065 China; 4 Department of Otolaryngology, Hua Dong Sanatorium, Wuxi, 214065 China; 5 Department of Biostatistics, School of Public Health, Suzhou Medical College of Soochow University, Suzhou, 215123 China; 6 Jiangsu Key Laboratory of Preventive and Translational Medicine for Geriatric Diseases, Suzhou Medical College of Soochow University, Suzhou, 215123 China

**Keywords:** Retrospective cross-sectional study, Carotid atherosclerosis, Fundus arteriosclerosis, Binary logistic regression, Retinal arterioles

## Abstract

**Objectives:**

Vascular stiffening is highly predictive of major adverse cardiovascular events. It is not clear whether microangiopathy, such as fundus arteriosclerosis, is related to carotid atherosclerosis. Hence, this study was designed to investigate the relationship between carotid atherosclerosis and fundus arteriosclerosis among individuals of different sexes in the Chinese health-examination population.

**Methods:**

This retrospective cross-sectional study involved 20,836 participants, including 13050 males and 7786 females. All participants underwent a detailed health examination, including medical history assessment, physical examination, assessment of lifestyle factors, fundus photography, Doppler ultrasound examination of the neck, and laboratory examinations. Two trained ophthalmologists analysed fundus arteriosclerosis based on fundus photographs, while carotid atherosclerosis was diagnosed using colour Doppler sonography of the neck. Binary logistic regression was used to analyse the relationship between carotid atherosclerosis and fundus arteriosclerosis.

**Results:**

In participants with fundus arteriosclerosis, the incidence of carotid atherosclerosis was higher than that of participants without fundus arteriosclerosis (52.94% vs. 47.06%). After adjustments for potential confounding factors, fundus arteriosclerosis was significantly associated with the risk of carotid atherosclerosis. The OR with 95% CI for fundus arteriosclerosis was 1.17 (1.02, 1.34) with *p* = 0.0262, and individuals who did not have fundus arteriosclerosis were used as a reference in the total population. Fundus arteriosclerosis was associated with the incidence of carotid atherosclerosis in males (*p* = 0.0005) but not in females (*p* = 0.0746).

**Conclusions:**

Fundus arteriosclerosis was closely associated with carotid atherosclerosis in the Chinese population. This association was found in males but not in females.

## Introduction

Cardiovascular disease has become the leading cause of disability and premature death worldwide [1]. By 2030, it is estimated that approximately 23.6 million people will die from cardiovascular diseases each year. The enormous and still increasing burden of cardiovascular diseases on individuals, families, and health-care systems underscores the urgent need for proactive prevention and more effective treatments [2].

Atherosclerosis is the main pathological process of most cardiovascular diseases [3] and is related to changes in the structure and function of microcirculation in the heart, brain, and retina [4–6]. In apparently healthy individuals, early detection of atherosclerosis is mainly focused on peripheral arteries and carotid arteries [7]. Carotid atherosclerosis may first present as thickening of the carotid intima–media layers, followed by fibrous fatty plaque and atheromatous plaque formation, narrowing the blood vessel lumen and weakening the walls. Next, it causes ischaemic cerebrovascular events through corresponding haemodynamic changes. There is substantial evidence that carotid endarterectomy (CEA) is beneficial for patients with severe carotid atherosclerosis as demonstrated by the European Carotid Surgery Trial (ECST) [8] and the North American Symptomatic Carotid Trial (NASCET) [9]. The network of blood vessels in the fundus is the only part of the human body, where the microcirculation can be observed directly, and the retinal vasculature can represent a potential indicator of a significant number of vascular and systemic diseases [10–12]. Fundus arteriosclerosis is essentially retinopathy and is a type of small arteriosclerosis. In addition, fundus arteriosclerosis has been shown to be closely associated with cardiovascular diseases, coronary artery disease, new-onset hypertension, atherosclerosis, and renal and cerebrovascular diseases in various populations [13–19]. Can ophthalmologists actively refer patients to the ultrasound department to undergo carotid ultrasonography for exclusion of carotid atherosclerosis? For patients with severe carotid artery stenosis (including asymptomatic cases), immediate treatments should be performed in the hope of reducing the incidence of cardiovascular and cerebrovascular diseases. However, there is still some controversy in the study of the relationship between carotid atherosclerosis and fundus arteriosclerosis [20, 27].

The purpose of this study was to evaluate the association between fundus arteriosclerosis and carotid atherosclerosis in the physical examination population among different sexes, possibly strengthening the recommendation for further carotid atherosclerosis screening for patients with retinal vascular disease.

## Methods

### Study population

**Fig. 1:**
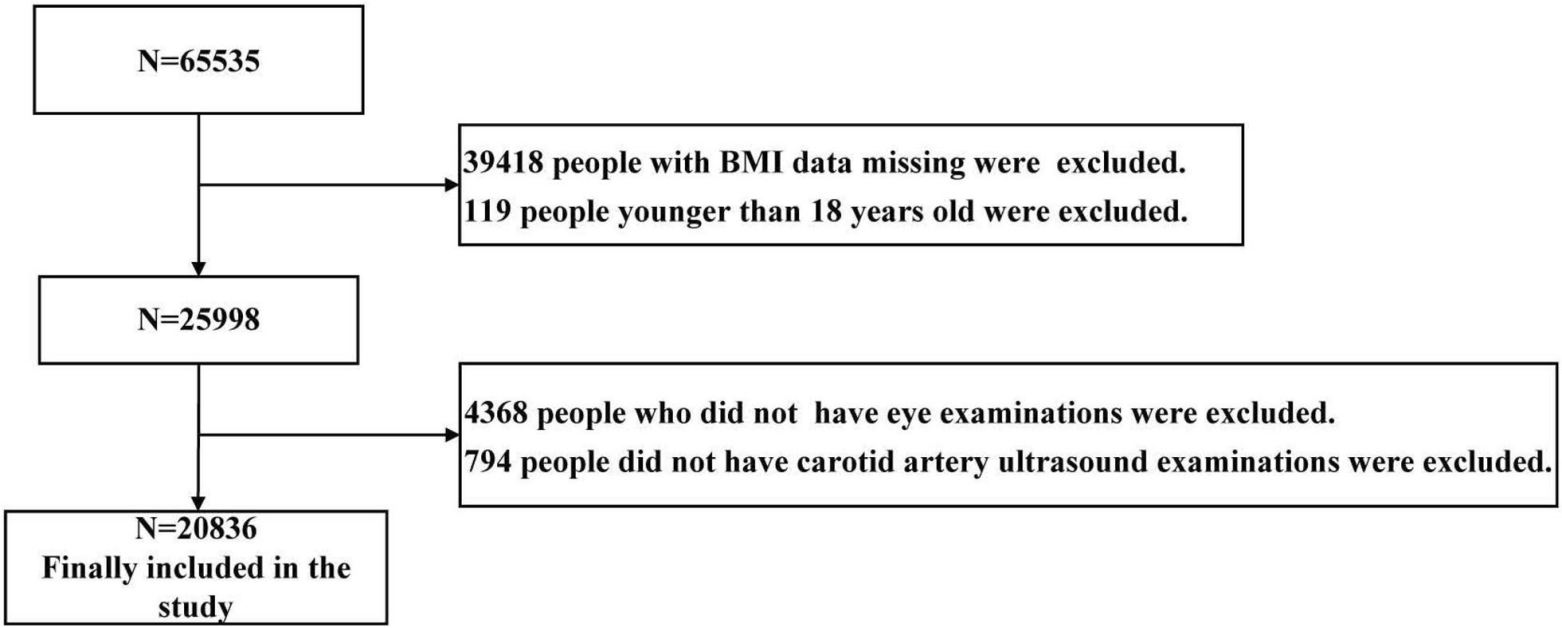
Description of the sample flow chart for screening studies for statistical analysis

This study analysed data from the Hua Dong Sanatorium. A total of 30,535 nonmanual workers underwent health examinations in 2018. The excluded population included those who had not undergone carotid ultrasound examination, had not undergone eye examination, had missing BMI values and were aged < 18 years. Finally, 20836 people (13050 males and 7786 females) aged 18–92 years were included in this study. The data cleaning steps are presented in Fig. 1.

This study was approved by the Ethical Committee and the Institutional Review Board of Hua Dong Sanatorium, Wuxi (No. 1, 2021). Informed consent was waived, and the need to waive informed consent was also supported by the Ethics Committee of Hua Dong Sanatorium. All methods were performed in accordance with the 1975 Declaration of Helsinki and the relevant guidelines.

### Questionnaire data

A standard questionnaire was administered by trained staff to obtain information about demographic characteristics (age, sex) and lifestyle risk factors, such as smoking and drinking. The interview included questions related to the diagnosis and treatment of diabetes, hypertension, and cardiovascular events. Smoking habits were categorized as current smoking, nonsmoking, and previous smoking. Drinking was categorized as current drinking, nondrinking, and previous drinking.

### Physical examination

Physical examinations, including weight, height, and blood pressure. When height and weight were measured, the patient stood upright, wearing a single layer of clothing without hat or shoes. Body mass index (BMI) was calculated as weight in kilograms divided by the square of height in metres (kg/m^2^). Blood pressure was measuring after 5 min of resting. Hypertension was defined as blood pressure ≥ 140/90 mmHg, a self-reported diagnosis of hypertension by a physician, or as the condition of an individual currently using antihypertensive drugs [28]^.^ Diabetes was defined as a fasting glucose level ≥ 7.0 mmol/L, a self-reported diagnosis of diabetes by a physician, or the condition of taking oral hypoglycaemic medication or insulin [29].

### Laboratory measurements

After a fast of at least 8 h, blood samples were collected from the anterior cubital vein in the morning. Fasting blood glucose levels were determined by a colorimetry method (AU5400, BECKMAN COULTER). Enzymatic methods were used to measure total cholesterol (TC), triglyceride (TG), low-density lipoprotein (LDL), and high-density lipoprotein (HDL) (AU5400, BECKMAN COULTER).

### Ultrasonography measurements

Each subject underwent carotid artery Doppler ultrasound examination. Carotid ultrasonography was performed by two experienced ultrasonographers under a standard protocol. Colour Doppler sonography was performed with GE Logic E9 (General Electric Company, USA).

All subjects were examined in a supine position with their heads turned 45 degrees from the site being scanned. The carotid arteries were examined bilaterally at the common.

carotid arteries, the bifurcation, the external carotid arteries, and the internal carotid arteries from transverse and longitudinal orientations and were scanned in the anterolateral, posterolateral, and mediolateral directions to assess the presence of atherosclerotic plaque and stenosis and measure IMT. The IMT was defined as the distance between the leading edge of the lumen–intima echo and the leading edge of the media–adventitia echo. The common carotid artery IMT was measured online in.

the posterior wall 10–20 mm proximal to the carotid bifurcation in a region free of focal plaque [30]. According to the Mannheim consensus [31], atherosclerotic plaques were defined as focal structures encroaching into the arterial lumen of 0.5 mm or 50% of the surrounding IMT value or IMT of > 1.5 mm. Carotid atherosclerosis was defined as the presence of atherosclerotic plaques in any of the aforementioned arterial segments [30].

### Ophthalmology measurement

The fundus camera examination of each subject was performed according to the standard protocol described in our previous study [32]. An eye exam was performed by a trained ophthalmologist. Fundus photography was completed following a standardized protocol [33]. All participants received fundus photography with a nonmydriatic method. Fundus photographs were acquired using a no-dilatation fundus camera (NW400, Topcon Corporation, Tokyo, Japan). The analysis of fundus arteries was evaluated by two trained ophthalmologists. Subjects were classified with regard to their retinal photography based on the Keith–Wagener–Barker classification [34]. Grade 1 is defined as retinal artery spasm or mild sclerosis. Grade 2 is defined as moderate to marked sclerosis of the retinal arterioles; the arteriovenous intersection can present different degrees of pathological changes, arteriosclerotic retinopathy or thrombosis of retinal veins. Grade 3 is defined as angiospastic retinopathy, characterized by oedema, cotton–wool patches, and haemorrhages in the retina, in addition to marked sclerosis of the retinal arterioles. Grade 4 is defined as measurable oedema of the disks in addition to grade 3 pictures [35]. In the present study, subjects without retinopathy were graded as normal, and subjects with any of the four grades were considered to have fundus arteriosclerosis.

### Statistical analyses

Baseline characteristics were compared in the total population with or without carotid atherosclerosis. Baseline characteristics of the participants were reported as medians (quartile intervals) for continuous variables (the continuous variables were not normally distributed) and numbers (percentages) for categorical variables. Kruskal–Wallis tests were employed to compare continuous variables, whereas categorical variables were compared by χ^2^ trend tests. Univariate and multivariate logistic regression analyses were used to estimate odds ratios (ORs) and 95% confidence intervals (CIs) for fundus arteriosclerosis in total populations, males and females.

To examine the consistency of the observed association between carotid atherosclerosis and fundus arteriosclerosis, we performed subgroup analyses of participants according to age, BMI (≤ 25, > 25 kg/m^2^), hypertension (yes, no), and diabetes (yes, no) in total, males and females. Statistical analyses were performed with SAS version 9.4 (SAS Institute, Cary, NC, USA). A two-sided *p* value < 0.05 was considered to be statistically significant.

## Results

### Characteristics of the study population

Continuous variables are expressed as the median (interquartile range); categorical variables are expressed as frequencies (percentages).

The baseline characteristics of the participants are shown in Table 1. The incidence of carotid atherosclerosis in males was higher than that in females (9.36% vs. 4.61%). Participants with carotid atherosclerosis tended to be older, had higher BMI, SBP, DBP, FBG, TG, TC, LDL, incidence of hypertension, diabetes and fundus arteriosclerosis, and had lower HDL than those without carotid atherosclerosis (Table 1).

**Table 1:** Study participants were categorized based on carotid atherosclerosis

Characteristics	Carotid atherosclerosis		*p* value
	Yes	No	
N (participants, %)	1581(7.59)	19255(92.41)	
Age, years	58(54, 64)	49(40, 55)	< 0.0001
Males, n (%)	1222(9.36)	11828(90.64)	< 0.0001
Females, n (%)	359(4.61)	7427(95.39)	
BMI, kg/m^2^	25.00(23.15, 26.91)	24.20(22.04, 26.41)	< 0.0001
SBP, mmHg	126(116, 137)	120(110, 130)	< 0.0001
DBP, mmHg	73(67, 80)	72(66, 80)	0.0007
FBG, mmol/L	5.73(5.32, 6.46)	5.45(5.14, 5.86)	< 0.0001
TG, mmol/L	1.63(1.15, 2.50)	1.44(0.95, 2.27)	< 0.0001
TC, mmol/L	5.09(4.44, 5.77)	5.02(4.46, 5.64)	0.0553
HDL, mmol/L	1.25(1.06, 1.52)	1.36(1.11, 1.66)	< 0.0001
LDL, mmol/L	3.13(2.56, 3.73)	3.03(2.53, 3.57)	< 0.0001
Hypertension, n (%)			< 0.0001
Yes	841(53.19)	4568(23.72)	
No	740(46.81)	14,687(76.28)	
Diabetes, n (%)			< 0.0001
Yes	323(20.43)	1532(7.96)	
No	1258(79.57)	17723(92.04)	
Fundus atherosclerosis, n (%)			< 0.0001
Yes	837(52.94)	3737(19.41)	
No	744 (47.06)	15518(80.59)	

*BMI* body mass index, *SBP* systolic pressure, *DBP* diastolic pressure, *FBG* fasting blood glucose, *TG* triglycerides, *TC* total cholesterol, *LDL* low density lipoprotein, *HDL* high density lipoprotein

### Relationship between fundus arteriosclerosis and the incidence of carotid atherosclerosis

As shown in Table 2, fundus arteriosclerosis was an independent risk factor for increasing carotid atherosclerosis incidence after adjustment for age, sex, BMI, smoking, drinking, SBP, DBP, FBG, diabetes, hypertension, TG, TC, LDL and HDL.

**Table 2:** Univariate and multivariate logistic regression analyses of the relationship between fundus arteriosclerosis and carotid atherosclerosis

Variables	N (participants)	Model 1		Model 2		Model 3	
		OR, 95% CI	*p* value	OR, 95% CI	*p* value	OR, 95% CI	*p* value
Total	20836						
No		1.00(refer)		1.00(refer)		1.00(refer)	
Yes		4.67(4.21, 5.19)	< 0.0001	1.33(1.16, 1.52)	< 0.0001	1.17(1.02, 1.34)	0.0262
Males	13050						
No		1.00(refer)	–	1.00(refer)	–	1.00(refer)	-
Yes		3.96(3.51, 4.46)	< 0.0001	1.48(1.28, 1.72)	< 0.0001	1.31(1.13, 1.53)	0.0005
Females	7786						
No		1.00(refer)	–	1.00(refer)	–	1.00(refer)	–
Yes		5.74(4.61, 7.15)	< 0.0001	0.88(0.65, 1.19)	0.3912	0.75(0.55, 1.03)	0.0746

*BMI* body mass index, *SBP* systolic pressure, *DBP* diastolic pressure, *FBG* fasting blood glucose, *TG* triglycerides, *TC* total cholesterol, *LDL* low density lipoprotein, *HDL* high density lipoprotein

Model 1: unadjusted

Model 2: adjusted for age, BMI, smoking, drinking, FBG, diabetes, and hypertension; there was no adjustment for sex in males and females

Model 3: adjusted for age, sex, BMI, smoking, drinking, FBG, diabetes, hypertension, TG, TC, LDL and HDL; there was no adjustment for sex in males and females

After adjusting for potential confounding factors, fundus arteriosclerosis was associated with an 17% higher likelihood (OR = 1.17 vs. no fundus arteriosclerosis, 95% CI  1.02–1.34) of carotid atherosclerosis in total population. For males, fundus arteriosclerosis was associated with the incidence of carotid atherosclerosis (OR = 1.31, 95% CI 1.13–1.53), but not in females (OR = 0.75, 95% CI 0.55–1.03). The above content has been added to the original text and marked in red text.

**Fig. 2:**
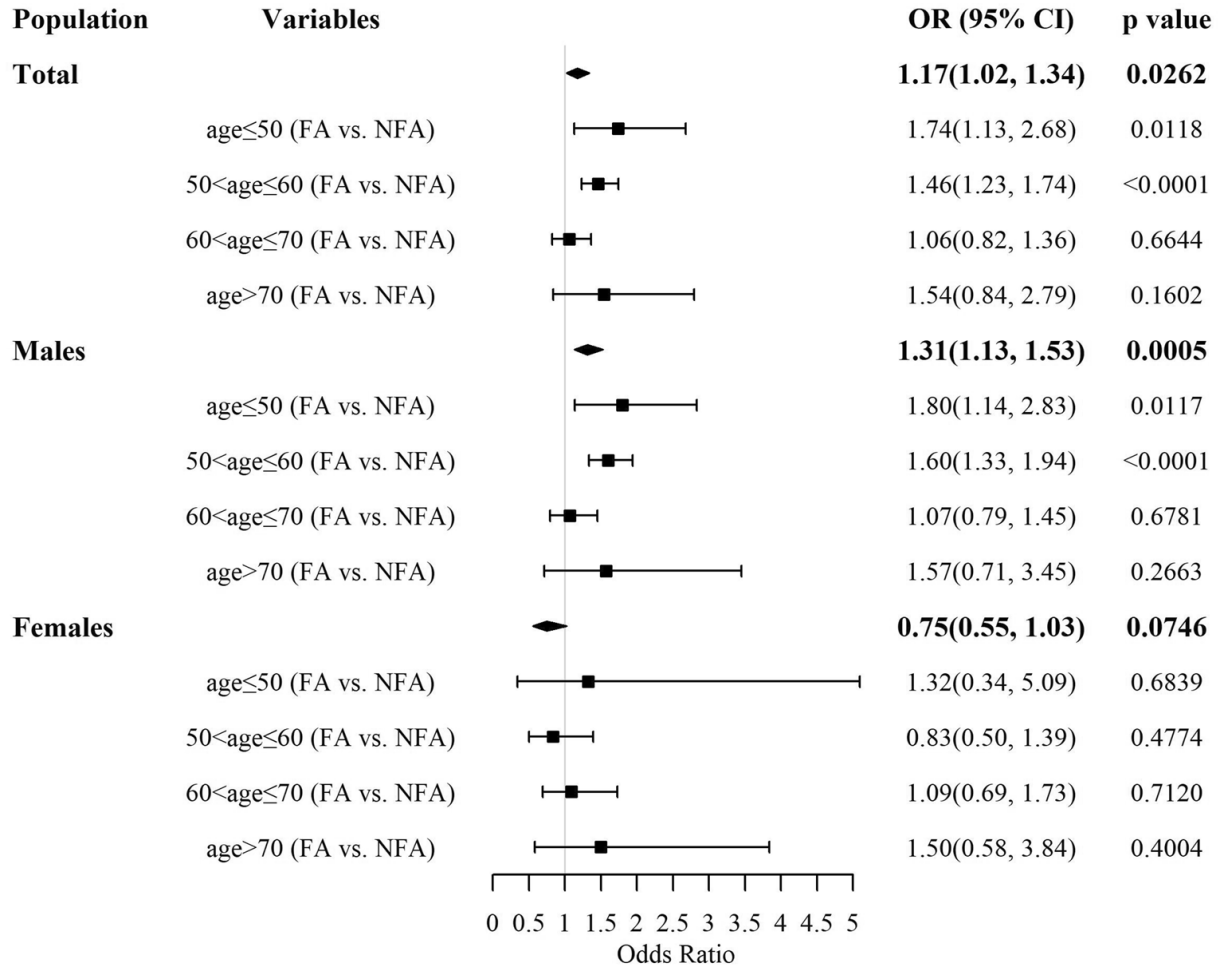
Relationship between fundus arteriosclerosis and carotid atherosclerosis in different populations of all ages FA: participants with fundus arteriosclerosis. NFA: participants without fundus arteriosclerosis

Figure 2 shows the relationship between fundus arteriosclerosis and carotid atherosclerosis during different ages. We found that there were statistically significant relationships between fundus arteriosclerosis and carotid atherosclerosis in age ≤ 50 years and 50 < age ≤ 60 years in total, after adjusting for gender, BMI, smoking, drinking, FBG, hypertension, diabetes, TG, TC, LDL and HDL. The OR with 95% CI for fundus arteriosclerosis were 1.74 (1.13, 2.68) with *p* = 0.0118 and 1.46 (1.23, 1.74) with *p* < 0.0001, respectively, people who without fundus arteriosclerosis as a reference. However, there were no statistically significant relationships between fundus arteriosclerosis and carotid atherosclerosis in 60 < age ≤ 70 years and age > 70 years in total. The OR with 95% CI for participants with fundus arteriosclerosis were 1.06 (0.82, 1.36) with *p* = 0.6644 and 1.54 (0.84, 2.79) with *p* = 0.1602, respectively, people who without fundus arteriosclerosis as a reference.

In males, there were statistically significant relationships between fundus arteriosclerosis and carotid atherosclerosis in age ≤ 50 years and 50 < age ≤ 60 years, after adjusting for BMI, smoking, drinking, FBG, hypertension, diabetes, TG, TC, LDL and HDL. The OR with 95% CI for fundus arteriosclerosis were 1.80 (1.14, 2.83) with *p* = 0.0117 and 1.60 (1.33, 1.94) with *p* < 0.0001, respectively, people who without fundus arteriosclerosis as a reference. However, there were no statistically significant relationships between fundus arteriosclerosis and carotid atherosclerosis in 60 < age ≤ 70 years and age > 70 years in males. The OR with 95% CI for participants with fundus arteriosclerosis were 1.07 (0.79, 1.45) with *p* = 0.6781 and 1.57 (0.71, 3.45) with *p* = 0.2663, respectively, people who without fundus arteriosclerosis as a reference. In addition, there was no relationship between fundus atherosclerosis and carotid atherosclerosis in females of all ages.

**Fig. 3:**
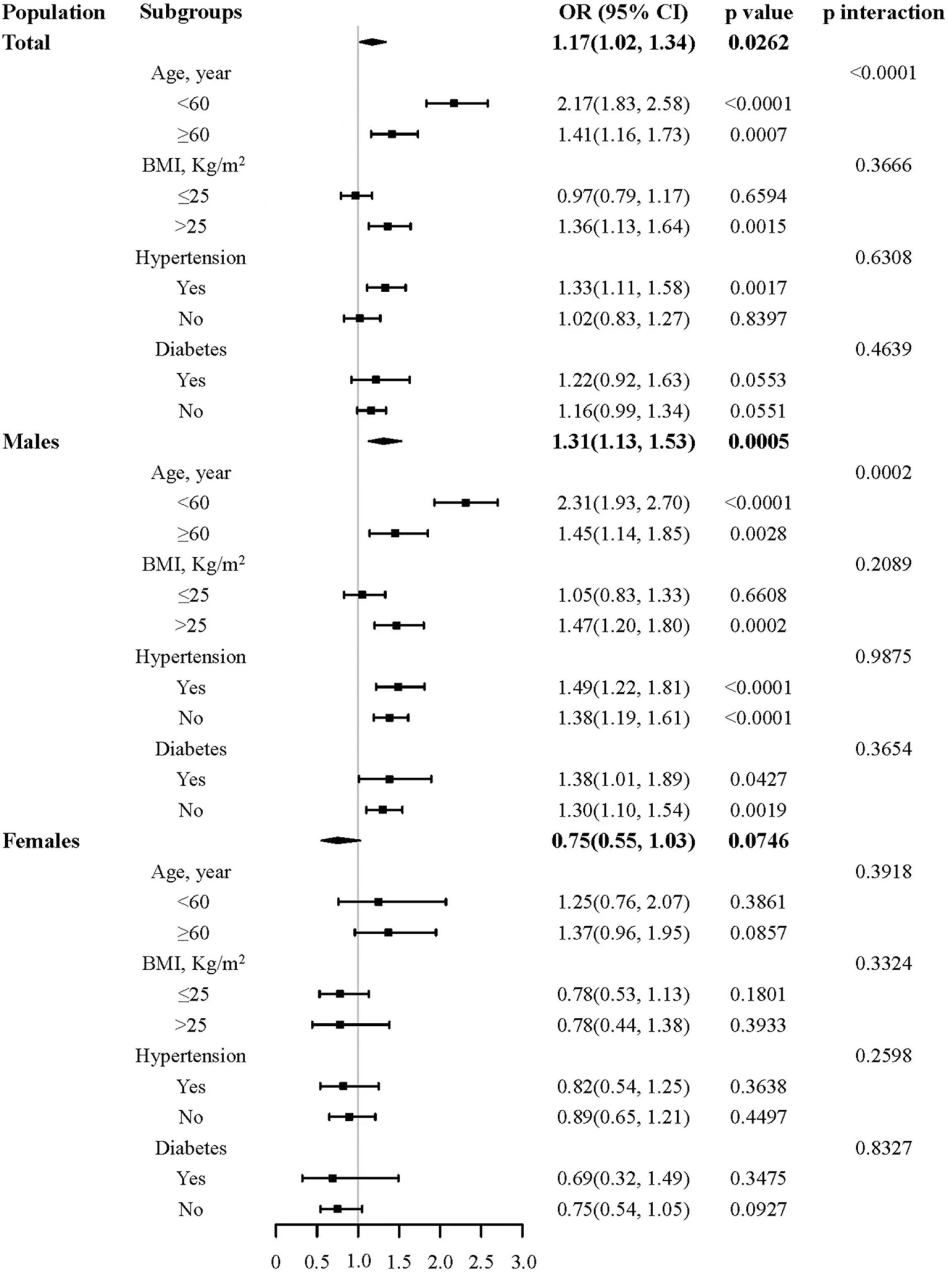
Subgroup analysis of the relationship between fundus arteriosclerosis and carotid atherosclerosis

Figure 3 shows in total with BMI ≤ 25 kg/m^2^, after adjusting for age, gender, smoking, drinking, FBG, hypertension, diabetes, TG, TC, LDL and HDL, there were no statistically associations between fundus arteriosclerosis and carotid arteriosclerosis, people who without fundus arteriosclerosis as a reference. The OR with 95% CI for fundus arteriosclerosis was 0.97 (0.79, 1.17) with *p* = 0.6594. In total with BMI > 25 kg/m^2^, after adjusting for age, gender, smoking, drinking, FBG, hypertension, diabetes, TG, TC, LDL and HDL, fundus arteriosclerosis was an independent risk factor for the incidence of carotid atherosclerosis, people who without fundus arteriosclerosis as a reference. The OR with 95% CI for fundus arteriosclerosis was 1.36 (1.13, 1.64) with *p* = 0.0015. Among males with BMI > 25 kg/m^2^, after adjusting for other confounding factors, fundus arteriosclerosis was an independent risk factor for carotid atherosclerosis, people who without fundus arteriosclerosis as a reference. The OR with 95% CI for fundus arteriosclerosis was 1.47 (1.20, 1.80) with *p* = 0.0002. However, there was no statistical association between fundus arteriosclerosis and carotid atherosclerosis in males with BMI < 25 kg/m^2^ and in females.

In the subgroup with hypertension, after adjusting for age, gender, BMI, smoking, drinking, FBG, diabetes, TG, TC, LDL and HDL, fundus arteriosclerosis was an independent risk factor for the incidence of carotid atherosclerosis, people who without fundus arteriosclerosis as a reference. The OR with 95% CI for fundus arteriosclerosis was 1.33 (1.11, 1.58) with *p* = 0.0017. In the subgroup without hypertension, after adjusting for age, gender, BMI, smoking, drinking, FBG, diabetes, TG, TC, LDL and HDL, there were no statistically associations between fundus arteriosclerosis and carotid atherosclerosis, people who without fundus arteriosclerosis as a reference. The OR with 95% CI for fundus arteriosclerosis was 1.02 (0.83, 1.27) with *p* = 0.8397. Among males with hypertension, after adjusting for other confounding factors, fundus arteriosclerosis was an independent risk factor for carotid atherosclerosis, people who without fundus arteriosclerosis as a reference. The OR with 95% CI for fundus arteriosclerosis was 1.49 (1.22, 1.81) with *p* < 0.0001. However, there was no statistical association between fundus arteriosclerosis and carotid atherosclerosis in males without hypertension and females.

In the subgroup with diabetes, after adjusting for age, gender, BMI, smoking, drinking, FBG, hypertension, TG, TC, LDL and HDL, there were no statistically associations between fundus arteriosclerosis and carotid atherosclerosis, people who without fundus arteriosclerosis as a reference. The OR with 95% CI for fundus arteriosclerosis was 1.22 (0.92, 1.63) with *p* = 0.0553. In the subgroup without diabetes, after adjusting for age, gender, BMI, smoking, drinking, FBG, hypertension, TG, TC, LDL and HDL, fundus arteriosclerosis was an independent risk factor for the incidence of carotid atherosclerosis, people who without fundus arteriosclerosis as a reference. The OR with 95% CI for fundus arteriosclerosis was 1.16 (0.99, 1.34) with *p* = 0.0551. Among males with and without diabetes, after adjusting for other confounding factors, fundus arteriosclerosis was an independent risk factor for carotid atherosclerosis, people who without fundus arteriosclerosis as a reference. The OR with 95% CI for fundus arteriosclerosis was 1.38 (1.01, 1.89) with *p* = 0.0427, 1.30 (1.10, 1.54) with *p* = 0.0019, respectively. However, there was no statistical association between fundus arteriosclerosis and carotid atherosclerosis in females.

The results of the subgroup analysis showed that there was a gender-specific association between fundus arteriosclerosis and carotid atherosclerosis. There was a statistical association in males, but not in females. In addition, there was no statistically significant relationship between the interaction of various subgroup variables with fundus arteriosclerosis and the incidence of carotid atherosclerosis (*p* interaction > 0.05 all).

## Discussion

In this large retrospective cross-sectional study from hospital medical examination data, we found that the incidence of carotid atherosclerosis in individuals with fundus arteriosclerosis was higher than that in individuals without fundus arteriosclerosis (18.30% vs. 4.58%). Our study demonstrated that fundus arteriosclerosis was significantly associated with carotid atherosclerosis. Moreover, we observed a particularly important result that there was a sex-specific association between fundus arteriosclerosis and carotid atherosclerosis. These findings provide pivotal evidence that fundus arteriosclerosis might increase carotid atherosclerosis risk and represent a potential therapeutic target in the primary prevention of carotid atherosclerosis. Moreover, it may be helpful in the early detection and early treatment of cardiovascular diseases.

Interestingly, in our study, there was a relationship between fundus arteriosclerosis and carotid atherosclerosis in males but not in females. This finding was consistent with previous results [36, 37]. In addition, this study showed that the incidence of carotid atherosclerosis in males was higher than that in females. One study has shown that the incidence of fundus atherosclerosis in males was higher than that in females in the Chinese population [32], which may also be the reason for the sex difference in the incidence of carotid atherosclerosis. This sex-specific association may be caused by different hormone levels. Sex-related disparities in atherosclerosis are likely attributed to variations in the influence of oestrogens and androgens on the disease. Androgens are thought to facilitate foam cell formation, the expression of atherogenic genes, and vascular endothelial cell apoptosis, thereby promoting atherosclerosis development. Conversely, oestrogens exert antiatherosclerotic effects by enhancing nitric oxide synthesis, vasodilation, and hyaluronan deposition and by inhibiting oxidative stress and vascular smooth muscle cell proliferation [38].

The relationship between fundus arteriosclerosis and carotid atherosclerosis was affected by age. This study showed that there was no significant statistical association between fundus atherosclerosis and carotid atherosclerosis in participants older than 60 years. In males younger than 60 years, the effect of fundus arteriosclerosis on carotid atherosclerosis was statistically significant. The results of this study suggest that we should promote assessment of the fundus in the physical examination of male individuals aged < 60 years. On one hand, the blood vessels in the fundus can be directly observed with the naked eye [12], and their pathological changes can directly reflect the pathological conditions of other systemic blood vessels [13, 16]. On the other hand, fundus examination is convenient and economical, and ophthalmologists should actively refer patients to the ultrasound department to undergo carotid ultrasonography for exclusion of carotid atherosclerosis. There was no relationship between fundus arteriosclerosis and carotid atherosclerosis in people aged > 60 years, which might be related to the high incidence of carotid atherosclerosis in this age group. In the 61–70-year-old group, the incidence of carotid atherosclerosis in individuals with fundus atherosclerosis and without fundus atherosclerosis was relatively close (21.1% vs. 17.4%). In the ≥ 71-year-old group, the incidence of carotid atherosclerosis in individuals with fundus atherosclerosis and without fundus atherosclerosis was also relatively close (39.8% vs. 29.0%). The incidence of carotid atherosclerosis was higher in individuals older than 60 years, and fundus arteriosclerosis had little effect on carotid atherosclerosis. This study did not find an association between fundus atherosclerosis and carotid atherosclerosis in individuals older than 60 years.

Some studies have shown that obesity is an independent risk factor for carotid atherosclerosis, especially in males younger than 70 years [39]. The results of our study are consistent with previous results. In individuals with BMI ≥ 25kg/m^2^, we found that there was an association between fundus arteriosclerosis and carotid atherosclerosis. There was no statistical relationship between the interaction of BMI and fundus arteriosclerosis and carotid atherosclerosis, and the interaction *p* value was 0.2955. However, the specific mechanism of the association between fundus atherosclerosis and carotid atherosclerosis found only in participants with BMI > 25 kg/m^2^ is still unclear, and more studies are needed to confirm this result. In addition, the results of this study suggested that we should pay more attention to fundus examinations in obese individuals undergoing physical examinations.

Moreover, the results of this study showed that in participants with hypertension or diabetes, there was a statistical association between fundus arteriosclerosis and carotid atherosclerosis. Previous studies have shown that diabetic retinopathy can increase the risk of carotid atherosclerosis [26], and vascular retinopathy has good predictive value in identifying asymptomatic carotid atherosclerosis [40]. Our research results were consistent with previous results. In addition, we found that there was a sex difference in this relationship, which was observed only in males and not in females. The mechanism of the association between fundus arteriosclerosis and carotid atherosclerosis in individuals with chronic diseases is not yet clear, and more studies are needed to confirm this. This study suggested that more attention should be given to ophthalmology examinations in individuals undergoing physical examinations for chronic diseases, especially males. This study provides a theoretical basis for the early detection and early diagnosis of cardiovascular disease.

There were still some limitations in this study. First, considering the cross-sectional design, we were unable to establish a causal relationship between fundus atherosclerosis and carotid atherosclerosis. Second, the relationship between disease and absence was based on doctors' records in this study, and no parameter measurement values of carotid atherosclerosis were involved. The results may not be accurate enough, and more research is needed to confirm them. In addition, our participants came from health examination personnel, the sample size was large, males and females were analysed separately to ensure sufficient parameters and accurate results, and a conclusion was drawn; that is, there were differences between males and females. Finally, further studies, including prospective investigations, are needed to determine whether fundus atherosclerosis can be used as an independent predictor of long-term carotid atherosclerosis and whether reducing fundus atherosclerosis can reduce the occurrence of carotid atherosclerotic events.

## Conclusions

In this study, we found a significant association between fundus arteriosclerosis and carotid atherosclerosis in the Chinese population. This association was particularly pronounced in males, suggesting a sex-specific difference in the relationship between these two conditions. The findings of this study highlight the importance of considering sex-specific factors when assessing the risk and progression of atherosclerosis-related diseases. Further research is needed to elucidate the underlying mechanisms and to develop targeted prevention and treatment strategies for individuals at risk. These results contribute to our understanding of the complex interplay between fundus and carotid artery health and their implications for cardiovascular health in the Chinese population.

## Data Availability

The raw data supporting the conclusions of this article will be made available by the authors without undue reservation.
